# Possible link between the apparently pathogenic *FANCI* variant and beneficial effects in sports performance

**DOI:** 10.3389/fgene.2026.1745694

**Published:** 2026-02-09

**Authors:** Mariusz Berdyński, Małgorzata Borczyk, Kinga Humińska-Lisowska, Michał Korostyński, Paweł Cięszczyk, Cezary Żekanowski

**Affiliations:** 1 Department of Neurogenetics and Functional Genomics, Mossakowski Medical Research Institute, Polish Academy of Sciences, Warsaw, Poland; 2 Laboratory of Pharmacogenomics, Department of Molecular Neuropharmacology, Maj Institute of Pharmacology, Polish Academy of Sciences, Krakow, Poland; 3 Faculty of Physical Education, Gdansk University of Physical Education and Sport, Gdansk, Poland

**Keywords:** elite athletes, Fanconi anemia, genetic predisposition, genomic variant, mutation

## Abstract

Athletic performance is a multifactorial trait influenced by both genetic and environmental factors. Evolutionary pressure can lead to seemingly contradictory effects of genetic mutations, and carriers of deleterious mutations may exhibit advantages. The objective of our exploratory study was to identify rare deleterious variants influencing athletic success. A total of 101 top-elite Polish athletes were recruited for the whole-genome sequencing analysis. We identified a variant in the *FANCI* gene (rs121918164, R1285*) in three unrelated top-elite athletes. This variant was absent in a large group of subjects from the same population: the national-elite athletes group (n = 890) who presented a lower level of sporting success and non-athletes (n = 1,009). Although, the R1285* *FANCI* variant is predicted to be damaging, we hypothesize that under certain conditions its carriers may have gained an advantage that facilitates success in sports. However, due to the limitations of the study, our observations require further investigation and validation in a larger and more diverse study groups.

## 1 Introduction

Athletic performance is a complex, multifactorial trait (Posthumus et al., 2016; Noble and Noble, 2023). Although success in certain sports often depends on the cultural, traditional, or economic system in which the athletes live, in some cases genetic factors allow success regardless of environmental conditions (Semenova et al., 2023; Peplonska et al., 2017). Evolutionary pressure often results in seemingly contradictory influences of genetic mutations on the phenotype, with well-known cases of phenylketonuria or sickle-cell anemia mutation carriers gaining advantages (Withrock et al., 2015). Rare mutations causally linked to monogenic diseases can sometimes be associated with exceptional athletic performance. For example, multi-medalist skier Eero Mäntyranta carried a rare mutation in the *EPOR* gene, which causes autosomal dominant erythrocytosis (Juvonen et al., 1991). This highlights that potentially deleterious variants may provide a strong advantage under specific conditions or at particular moments in an athlete’s life, even if they may appear harmful under other circumstances.

The objective of our study was to identify rare deleterious variants potentially influencing athletic success. We identified a putatively deleterious variant in the *FANCI* gene (p.Arg1285*) in 101 Polish top-elite athletes. Furthermore, we investigated whether this variant is also present in groups of 890 national-elite athletes and 1,009 sedentary controls.

The *FANCI* gene (also known as *KIAA1794* and *FLJ10719)* encodes the Fanconi anemia (FA) complementation group I (FANCI) protein. FANCI is a component of the FA pathway that is essential for repairing interstrand cross-links (ICLs). The FANCI protein is a part of the FA complex and plays a crucial role in the cellular response to DNA ICLs. If left unrepaired, ICLs can lead to DNA double-strand breaks (DSBs). The FA repair pathway is a complex process involving 22 identified FA proteins (FANCA–FANCW) and several FA-associated proteins such as FAAP24 and FAAP100. FANCM recognizes DNA damage sites due to ICLs and recruits FA core complex proteins, which leads to the formation of a monoubiquitinated FANCI–FANCD2 heterodimer. Monoubiquitination of the heterodimer is the hallmark of FA pathway activation. The monoubiquitinated FANCI–FANCD2 heterodimer then recruits downstream repair factors, including SLX4 (FANCP), XPF (FANCQ), BRCA1 (FANCS), BRCA2 (FANCD1), and RAD51 (FANCR), to complete the ICL repair. Biallelic pathogenic variants in the *FANCI* gene cause FA. FA is an autosomal recessive or X-linked disorder (22 genes described as FA genes) characterized by congenital anomalies, defective hematopoiesis, and an increased risk of developing acute myeloid leukemia and certain solid tumors due to a deficiency in DNA repair, which results in a reduced ability to remove DNA ICLs (Kook, 2005; del Valle et al., 2020; Sondalle et al., 2019). Heterozygous mutations in FA-related genes (e.g., *BRCA1*, *BRCA2*, *BRIP1*, *PALB2*, and *RAD51C*) are associated with hereditary breast and/or ovarian cancer predisposition. However, results regarding cancer predisposition for the remaining FA-related genes, including *FANCI*, are inconclusive (del Valle et al., 2020; Daly et al., 2017).

## 2 Materials and methods

The study included 101 top-elite Polish male athletes (age 23.5 ± 5.9 years) who were ranked among the top 100 worldwide, had won medals in national championships, or had participated in European or World Championships (Supplementary Table S1). Based on the previous methodology (Dzitkowska-Zabielska et al., 2022), athletes were divided into speed (n = 53) and endurance (n = 48) groups (Supplementary Table S1). Whole-genome sequencing (WGS) was performed as previously described (Fichna et al., 2021). The *FANCI* rs121918164 variant frequency was screened using the TaqMan method (Supplementary File) in 890 national-elite athletes and 1,009 sedentary controls (Fichna et al., 2021).

The presence of the variant of interest was identified in three samples via WGS and confirmed via Sanger sequencing (Supplementary File 1).

### 2.1 Variant selection

A pre-selected gene list was used to identify deleterious variants of interest (Supplementary File 1). The gene list included genes enriched in human skeletal muscle tissue based on The Human Protein Atlas (https://www.proteinatlas.org/) (Uhlén et al., 2015), genes associated with improved performance and strength in mice, and genes linked to selected HPO terms. Variants in these genes were filtered to retain loss-of-function (LoF) variants. Deleteriousness was defined as stop-gain mutations with minor allele frequency (MAF) <0.05% (Supplementary File 1).

### 2.2 Principal component analysis (PCA)

Genotyping of athletes’ gVCF files was performed using the GATK GenotypeGVCFs v4.1.7.0 to call variants present in the 1000 Genomes Project Phase 3. After filtering, a total of 76,994,916 SNPs remained. PCA was performed on the athlete samples in combination with samples from the 1000 Genomes Project using the Hail library (https://hail.is/) on a randomly selected 0.01% (76,496) of all genotyped SNPs. The PCA was performed with Hardy–Weinberg normalization using the HWE-normalized PCA function implemented in Hail.

### 2.3 Fisher’s exact test

Fisher’s exact test was used to compare the rs121918164 variant in our cohort to European non-Finnish gnomAD controls (56 carriers and 589,929 non-carriers). For the Bonferroni correction, we adopted a conservative approach by assuming that we analyzed all high-confidence LoF variants as reported that were detected in a sample of 141,456 humans (Karczewski et al., 2020).

### 2.4 Probability calculations

The probability of a random occurrence of a rare variant was calculated according to the following formula:

$$P=\text{NSNPs }x\;{\left(\text{MAF}\right)}^{3}\;x\text{ Ntrios},$$

where NSNPs is the estimated number of possible SNPs within protein-coding genes (20,000 genes x 26,288 bp median gene length), MAF represents MAF from the non-Finnish European subset in gnomAD (0.00005) (date: 29.02.2024) (Lek et al., 2016), and Ntrios is the number of possible trios (161,700).

### 2.5 Linkage disequilibrium

For variants within 100 kb of the detected FANCI variant that had at least three non-reference calls in the whole-genome dataset, linkage disequilibrium (LD) was calculated with the LD() function from the Gaston R library (v 1.6) and plotted in a form of LD heatmap.

## 3 Results and discussion

Screening the genomes of 101 Polish top-elite athletes resulted in the identification of the potentially pathogenic variant (NM_001113378.2:c.3853C>T, NP_001106849.1:p.Arg1285*, rs121918164) in the *FANCI* gene in three athletes (two from the speed group and one from the endurance group) (Supplementary File 1 for details). The variant was absent in a database of healthy Polish individuals (n = 1,222; The Thousand Polish Genomes‐Nasze Genomy database) (Kaja et al., 2022). The variant is present in the Genome Aggregation Database (gnomAD); date: 13.02.2024) with an MAF of 0.00004276 with the highest prevalence in the South Asian (0.00009881) and European (non-Finnish) (0.00004746) subsets (Chen et al., 2024).

We analyzed the frequency of the variant using the TaqMan method. The variant was absent in national-elite Polish athletes (n = 890) and sedentary controls (n = 1,009) from the Polish population. It should be noted that the national-elite athlete group presented a lower level of competitive success than the initial group of international top-elite athletes.

The three top-elite athletes were born in distinct regions of Poland, far from each other, and were not related. A kinship analysis was conducted using the WGS data, and the results were confirmed using the PC-relate method implemented in Hail. The analysis confirmed that the individuals were unrelated. The highest kinship value among the tested pairs was 0.0026 (on a scale of 0–0.5) (Table 1).

**TABLE 1:** Kinship analysis based on the WGS results of all three individuals.

Sample A	Sample B	Kinship		
		All variants	Variants with an MAF >0.1	Variants with an MAF >0.05
Sample 1	Sample 2	0,00272	0,00282	0,00314
Sample 1	Sample 3	0,00046	0,00045	0,00038
Sample 2	Sample 3	0,00262	0,00276	0,00283

All other variants in the vicinity of the detected variant were analyzed, and two intergenic variants (rs974948748 and rs750419590) were found to be in perfect LD with the variant of interest. This suggests that the variant in question may be part of a specific regional haplotype (Supplementary Figure S3). These variants were not detected in a database of ∼1,000 Polish genomes and have MAFs of 0.000073 and 0.00018, respectively, in the non-Finnish European subset in gnomAD v 4.0.0.

We also performed an estimation of the false discovery rate (FDR). The probability of a random occurrence of a rare variant in the three selected samples (with MAF = 0.00005) was estimated to be 6.56*10^−8^. The probability of identifying such a rare variant in any trio from the group of 100 samples is approximately 1%. This 1% value can be interpreted as an estimated FDR for a coincidental finding. Fisher’s exact test comparing athletes to the non-Finnish European population yielded a p-value of 1.57 × 10^−7^, which is just above the genome-wide significance threshold for a full GWAS. In our study, we considered only the high-confidence LoF variants. Large-scale studies have detected 443,769 such high-confidence predicted LoF variants in humans; therefore, the Bonferroni-adjusted p-value for this variant would be 0.069 (Uhlén et al., 2015).

This estimated corrected p-value is likely higher than the actual corrected p-value as we adopted a very conservative approach. Since many of the LoF variants are very rare, a pool of 100 WGS samples is unlikely to contain as many of them as a dataset of over 100,000 WGS samples. As an example, an analysis of 60,000 human genomes revealed 179,774 high-confidence LoF variants (Karczewski et al., 2020). If we take this number as the estimate, the Bonferroni-adjusted p-value would be 0.028.

Given the extremely low frequency of the identified variant, a robust statistical assessment remains methodologically challenging. Nevertheless, we estimated that the probability of randomly identifying a triplet of such rare variants is exceptionally low (FDR < 1%). Additionally, the calculated overall p-value for the association marginally exceeds the conventional genome-wide significance threshold.

The rs121918164 *FANCI* variant is annotated as pathogenic in the clinical databases ClinVar (pathogenic/likely pathogenic) and LOVD (affects function/not classified) with deleterious consequences on the FANCI protein. It has been identified in a compound heterozygous state in patients with FA, in a patient with a Li–Fraumeni-like phenotype with breast cancer, and in a two-year-old boy with severe transfusion-dependent anemia and unstable Hakkari hemoglobin (Dorsman et al., 2007; Penkert et al., 2018; Mann et al., 2022).

A similar variant in the *FANCI* gene, c.3854G>A (p.Arg1285Gln), identified in close proximity, involves a nucleotide change at the second position within the same codon as the c.3853C>T (p.Arg1285*) variant. This underscores the potential for complex effects on protein structure and function. The c.3854G>A variant is predicted to create an additional ATM/ATR phosphorylation motif, suggesting that alterations in these pathways could significantly affect the efficacy of the DNA damage response (DDR). The DDR is essential for maintaining genomic integrity. Key regulators, including ATM, ATR, and DNA-PKcs, orchestrate repair processes and cellular processes to DNA damage, such as DSBs and replication stress (Dorsman et al., 2007).

It has also been shown that another mutation at the same codon (p.Arg1285Gly) appears to cause a milder phenotype in homozygosity (Savage et al., 2016). An *in vitro* study of BD0952 cells (lymphocytes from a patient with a classic FA presentation) shows that the *FANCI* mutation at the highly conserved Arg1285 at the C-terminus of the protein position (Arg1285Gln) causes FA in donor cells, makes the cells MMC-sensitive, and disturbs FANCD2 regulation (Smogorzewska et al., 2007). These results may indicate that the pathogenicity of the p.Arg1285* mutation depends on the genomic context and the contribution of other variants.

Despite potential harm, the presence of the p.Arg1285* mutation in three unrelated top-elite athletes may suggest both negative and, in some cases, positive effects on the phenotypes of the carrier. This phenomenon of seemingly contradictory (negative–positive) influences of genetic variants on the phenotype is well-recognized, with examples including sickle-cell anemia and malaria resistance and the *CCR5* mutation and HIV resistance (Withrock et al., 2015). The case of Eero Mäntyranta also showed that carrying a specific variant in the erythropoietin receptor (*EPOR*) gene, which causes autosomal dominant erythrocytosis-1, is potentially beneficial for endurance sports, without causing obvious adverse clinical symptoms (Juvonen et al., 1991). The spread of pathogenic mutations in populations may be explained by natural selection, which favors traits that are useful in specific situations, rather than the overall health or longevity of carriers (Gluckman et al., 2011).

The identification of the p.Arg1285* variant in the *FANCI* gene in elite Polish athletes raises an interesting question about the relationship between DNA damage repair mechanisms and athletic performance. Further studies are needed to elucidate its risk–benefit mechanism. There is a risk that *in vitro* or *in vivo* model studies may not capture subtle cellular-level effects that could contribute to a larger physiological outcome, which may be observable only within a specific time window.

We are unaware of any specific biological associations that could limit the possible positive effects of the variant to a certain time window‐such as the optimal age for training‐after which any evolutionary advantage may diminish or even become detrimental. In such a case, apart from direct observation of the variant (or variants) in larger cohorts and different populations, we may not have another feasible method of assessing its impact since the biological effect may not be detectable at the cellular or model organism level.

## 4 Limitations of the study

As we did not detect the variant in national-elite athletes and sedentary controls, we cannot report the exact or the corrected p-value for the detected variant. Despite multiple efforts to exclude the coincidental detection, including calculating the probability of a completely random finding, we cannot completely exclude the possibility that the variant was identified by chance in three unrelated top-elite athletes.

We cannot distinguish whether the p.Arg1285* variant is a causative or beneficial factor for the elite athlete phenotype or whether it is only associated with a specific haplotype or a nearby causative variant.

No medical or physiological data are available; health status cannot be assessed in this study. Therefore, we cannot study the negative or positive impact of the mutation on the physiological phenotype. However, since the variant carriers are athletes, and therefore physically fit, it can be assumed that they did not experience any adverse health effects, at least until they participated in the study.

## Data Availability

The raw data supporting the conclusions of this article will be made available by the authors, without undue reservation.

## References

[B1] Chen S. Francioli L. C. Goodrich J. K. Collins R. L. Kanai M. Wang Q. (2024). A genomic mutational constraint map using variation in 76,156 human genomes. Nature 625 (7993), 92–100. 10.1038/s41586-023-06045-0 38057664 PMC11629659

[B2] Daly M. B. Pilarski R. Berry M. Buys S. S. Farmer M. Friedman S. (2017). NCCN guidelines insights: genetic/familial high-risk assessment: breast and ovarian, version 2.2017. J. Natl. Compr. Canc Netw. 15 (1), 9–20. 10.6004/jnccn.2017.0003 28040716

[B3] del Valle J. Rofes P. Moreno-Cabrera J. M. López-Dóriga A. Belhadj S. Vargas-Parra G. (2020). Exploring the role of mutations in Fanconi Anemia Genes in hereditary cancer patients. Cancers (Basel) 12 (4), 829. 10.3390/cancers12040829 32235514 PMC7226125

[B4] Dorsman J. C. Levitus M. Rockx D. Rooimans M. A. Oostra A. B. Haitjema A. (2007). Identification of the Fanconi Anemia Complementation Group I gene, *FANCI* . Anal. Cell. Pathol. 29 (3), 211–218. 10.1155/2007/151968 17452773 PMC4618213

[B5] Dzitkowska-Zabielska M. Bojarczuk A. Borczyk M. Piechota M. Korostyński M. Adamczyk J. G. (2022). Transmission distortion of MCT1 rs1049434 among Polish elite athletes. Genes 13 (5), 870. 10.3390/genes13050870 35627255 PMC9142056

[B6] Fichna J. P. Humińska-Lisowska K. Safranow K. Adamczyk J. G. Cięszczyk P. Żekanowski C. (2021). Rare variant in the SLC6A2 encoding a norepinephrine transporter is associated with elite athletic performance in the Polish population. Genes (Basel) 12 (6), 919. 10.3390/genes12060919 34203885 PMC8232774

[B7] Gluckman P. D. Low F. M. Buklijas T. Hanson M. A. Beedle A. S. (2011). How evolutionary principles improve the understanding of human health and disease. Evol. Appl. 4 (2), 249–263. 10.1111/j.1752-4571.2010.00164.x 25567971 PMC3352556

[B8] Juvonen E. Ikkala E. Fyhrquist F. Ruutu T. (1991). Autosomal dominant erythrocytosis caused by increased sensitivity to erythropoietin. Blood 78 (11), 3066–3069. 10.1182/blood.v78.11.3066.bloodjournal78113066 1954391

[B9] Kaja E. Lejman A. Sielski D. Sypniewski M. Gambin T. Dawidziuk M. (2022). The thousand Polish Genomes-A database of Polish variant allele frequencies. Int. J. Mol. Sci. 23 (9), 4532. 10.3390/ijms23094532 35562925 PMC9104289

[B10] Karczewski K. J. Francioli L. C. Tiao G. Cummings B. B. Alföldi J. Wang Q. (2020). The mutational constraint spectrum quantified from variation in 141,456 humans. Nature 581 (7809), 434–443. 10.1038/s41586-020-2308-7 32461654 PMC7334197

[B11] Kook H. (2005). Fanconi anemia: current management. Hematology. 10 (Suppl. 1), 108–110. 10.1080/10245330512331390096 16188650

[B12] Lek M. Karczewski K. J. Minikel E. V. Samocha K. E. Banks E. Fennell T. (2016). Analysis of protein-coding genetic variation in 60,706 humans. Nature 536 (7616), 285–291. 10.1038/nature19057 27535533 PMC5018207

[B13] Mann S. G. Мс Г. Krasilnikova M. V. Км В. Karamjan N. A. Кн А. (2022). Rare unstable hemoglobin Hakkari in Russia: a case report and literature review. Pediatr. Hematology/Oncology Immunopathol. 21 (3), 77–83. 10.24287/1726-1708-2022-21-3-77-83

[B14] Noble R. Noble D. (2023). Understanding living systems. Cambridge: Cambridge University Press. Available online at: https://www.cambridge.org/core/books/understanding-living-systems/F528C289CA656D7C0F67DA5584E50611.

[B15] Penkert J. Schmidt G. Hofmann W. Schubert S. Schieck M. Auber B. (2018). Breast cancer patients suggestive of Li-Fraumeni syndrome: mutational spectrum, candidate genes, and unexplained heredity. Breast Cancer Res. 20 (1), 87. 10.1186/s13058-018-1011-1 30086788 PMC6081832

[B16] Peplonska B. Adamczyk J. G. Siewierski M. Safranow K. Maruszak A. Sozanski H. (2017). Genetic variants associated with physical and mental characteristics of the elite athletes in the Polish population. Scand. J. Med. Sci. Sports 27 (8), 788–800. 10.1111/sms.12687 27140937

[B17] Posthumus M. Caine D. J. Collins M. Hills A. P. Noakes T. Borms J. (2016). Genetics and sports. 2nd, revised and extended edition Basel Freiburg: Karger. 123.

[B18] Savage S. A. Ballew B. J. Giri N. NCI DCEG Cancer Genomics Research Laboratory, Chandrasekharappa S. C. Ameziane N. (2016). Novel FANCI mutations in Fanconi anemia with VACTERL association. Am. J. Med. Genet. A 170A (2), 386–391. 10.1002/ajmg.a.37461 26590883 PMC7158112

[B19] Semenova E. A. Hall E. C. R. Ahmetov I. I. (2023). Genes and Athletic Performance: the 2023 update. Genes 14 (6), 1235. 10.3390/genes14061235 37372415 PMC10298527

[B20] Smogorzewska A. Matsuoka S. Vinciguerra P. McDonald E. R. Hurov K. E. Luo J. (2007). Identification of the FANCI protein, a monoubiquitinated FANCD2 paralog required for DNA repair. Cell 129 (2), 289–301. 10.1016/j.cell.2007.03.009 17412408 PMC2175179

[B21] Sondalle S. B. Longerich S. Ogawa L. M. Sung P. Baserga S. J. (2019). Fanconi anemia protein FANCI functions in ribosome biogenesis. Proc. Natl. Acad. Sci. 116 (7), 2561–2570. 10.1073/pnas.1811557116 30692263 PMC6377447

[B22] Uhlén M. Fagerberg L. Hallström B. M. Lindskog C. Oksvold P. Mardinoglu A. (2015). Proteomics. Tissue-based map of the human proteome. Science 347 (6220), 1260419. 10.1126/science.1260419 25613900

[B23] Withrock I. C. Anderson S. J. Jefferson M. A. McCormack G. R. Mlynarczyk G. S. A. Nakama A. (2015). Genetic diseases conferring resistance to infectious diseases. Genes Dis. 2 (3), 247–254. 10.1016/j.gendis.2015.02.008 30258868 PMC6150079

