# Supplementary material for: Possible link between the apparently pathogenic FANCI variant and beneficial effects in sports performance
Source: Front Genet. 2026 Feb 9;17:1745694. doi: 10.3389/fgene.2026.1745694 (PMC12925683; doi:10.3389/fgene.2026.1745694)
Supplement: Supplementary file 1 [file DataSheet1.pdf]

Supplementary Material

1 Supplementary Data

Supplementary Table 1. Characteristics of groups of athletes by discipline and metabolic requirements.

Sports	N
**Speed**	**53**
sprints (100–400 m)	*27*
weightlifting	*24*
powerlifting	*2*
**Endurance:**	**48**
marathon	*7*
cross-country skiing	*16*
swimming (400–1500 m)	*22*
Triathlon	*2*
Rowing	*1*
**Total**	**101**

Variant selection process

The gene list included genes enriched in human skeletal muscle tissue based on the Human Protein Atlas (https://www.proteinatlas.org/)), genes associated with improved performance and strength in mice, and genes linked to selected HPO terms. Variants in these genes were filtered to retain extremely rare Loss of Function (LoF) variants. Deleteriousness was defined as stop-gain mutations with MAF < 0.05% (Table 1). No LoF homozygotes were detected. The majority of variants (13/22) were exclusively present in controls and absent in athletes, with most classified as high-confidence LoF according to the gnomAD database. Variants were additionally annotated with CADD scores - values derived from a machine learning algorithm that predicts deleteriousness (Rentzsch et al., 2019). CADD scores are Phred-scaled, with scores ≥30 indicating that only 0.1% of variants are predicted to be more deleterious. Most variants had high CADD scores (>30), indicating strong deleterious effects. Two mutations were found in multiple elite athletes: a low-confidence LoF variant in SMARCA2 with low predicted deleteriousness, and a high-confidence protein-truncating variant in the FANCI gene (Supplementary Table2).

Confirmation of Variant Presence and Genotyping Methodology

The presence of the variant identified by WGS method, was confirmed in three samples by Sanger sequencing using primers: GCGTGCTTGCTTTAGGTAGA and AAGCCAGCACTGACCTCTTC.

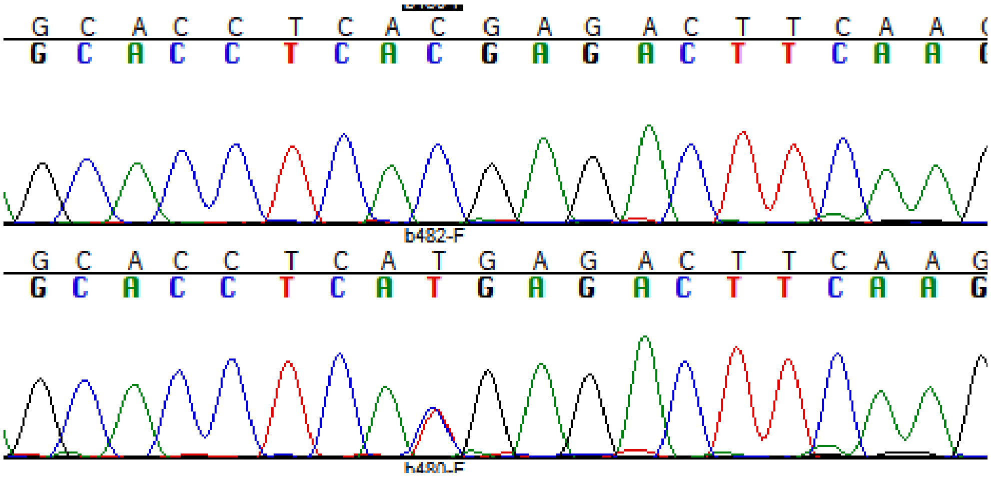

2 **Supplementary Figure 1. An electrochromatogram showing a heterozygous c.3853C>T mutation**

Genotyping of selected SNP was performed using TaqMan SNP genotyping assays (Assay ID: FANCI rs121918164 C_163475627_10, (Life Technologies, Carlsbad, CA, USA)

Supplementary Table 2. Rare variant analysis results. Variants with MAF < 0.05% labelled as stop-gain were selected.

contig	position	alleles	rsid	MAF	predicted LoF	cadd	gene	controls n=1222	athletes n=101
chr1	152311827	['G', 'C']	rs200360684	0.0001	end truncation	36	FLG, FLG-AS1	0	1
chr2	19941796	['A', 'C']	rs199952377	0.0004	high confidence	40	WDR35	1	0
chr2	32250936	['G', 'A']	rs199475953	0.0003	high confidence	31	NLRC4	0	1
chr2	178630250	['G', 'A']	rs140743001	1.5E-05	high confidence	58	TTN, TTN-AS1	1	0
chr3	136333472	['G',	rs13902	7E-05	high	4.46	PCCB	1	0

		'A']	24413		confidence				
chr5	148065358	['A', 'T']	rs1482249008	1.5E-05	high confidence	36	SPINK5	1	0
chr6	51619279	['G', 'C']	rs143616240	0.0001	end truncation	38	AL355997.1, PKHD1	2	0
chr7	56015124	['C', 'A']	rs1410416350	4.6E-05	high confidence	39	PSPH	0	1
chr7	117587778	['G', 'T']		0.0004	high confidence	40	CFTR	1	0
chr7	142773993	['G', 'T']		1.5E-05	high confidence	34	PRSS2	3	0
chr8	100233436	['C', 'T']	rs201740530	0.0002	nd	38	SPAG1	0	1
chr8	132887497	['G', 'T']	rs1282455611	1.5E-05	high confidence	35	TG	1	0
chr9	2161733	['T', 'G']		0.0005	Low-confidence	0.456	SMARCA2	0	2
chr9	104855973	['G', 'T']		0.0003	end truncation	3.44	ABCA1	0	1
chr12	38320937	['G', 'A']		1.5E-05	high confidence	41	ALG10B	1	0
chr12	88083161	['G', 'A']		6.2E-05	high confidence	42	CEP290	0	1
chr15	30905592	['C', 'G']	rs201220536	0.00035	high confidence	32	FAN1	1	0
chr15	89315318	['C', 'T']	rs121918164	9E-05	high confidence	36	FANCI, POLG	0	3
chr16	16202045	['G', 'A']	rs72650699	6E-05	Low-confidence	37	ABCC6	1	0
chr17	80048602	['C', 'T']	rs200710012	0.0002	Low-confidence	0.095	CCDC40	1	0
chr19	1244382	['C',		0.000	end	23.8	ATP5F	1	1

		'T']		3	truncation		1D		
chr22	50249399	['G', 'A']	rs10372 40982	3E-05	high confidence	35	HDAC 10,MA PK12	0	1

Principal Component Analysis (PCA):

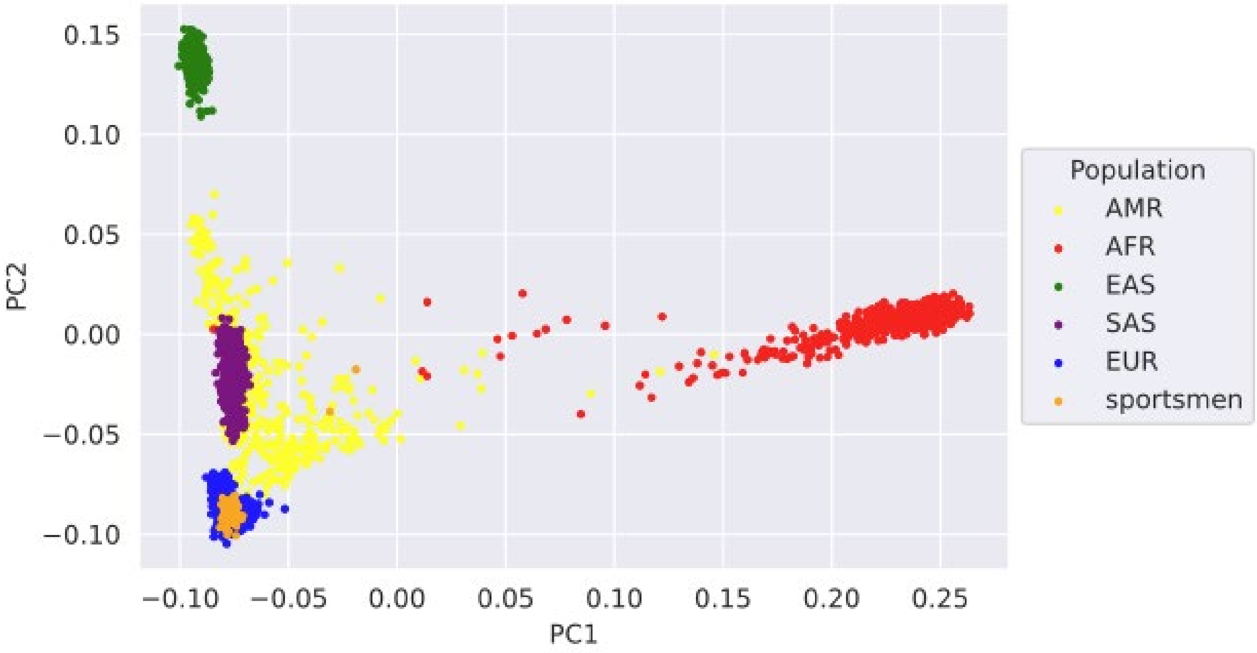

Supplementary Figure 2. PCA comparing a random subset of genotypes between Polish sportsmen and 1000 Genomes Project samples

Supplementary Figure 2. Linkage disequilibrium (LD) heatmap of sequenced variants in the vicinity of discovered FANCI stop-gain variant.

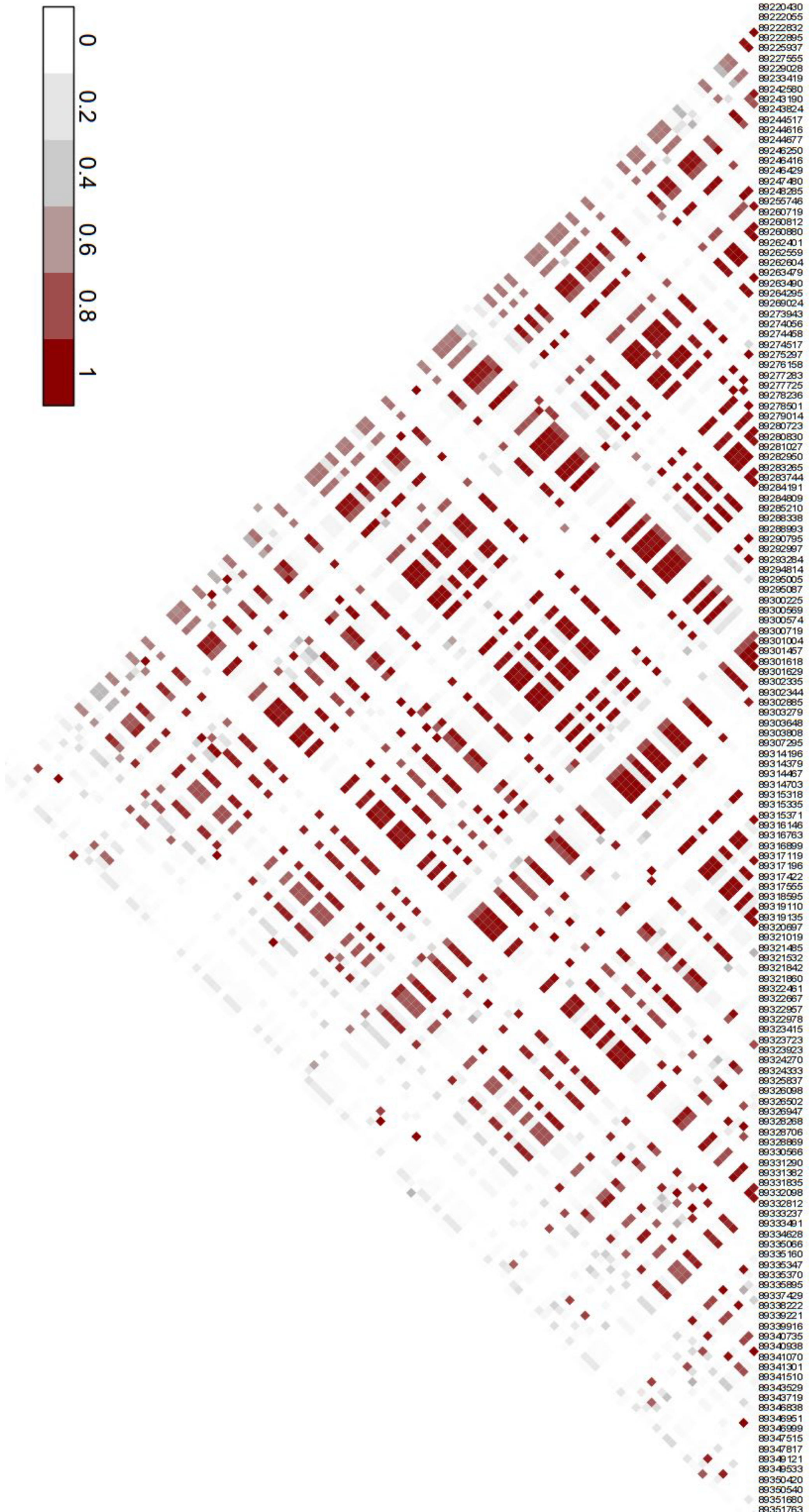
0
0.2
0.4
0.6
0.8
1
89220430
89222055
89222832
89222895
89225937
89227555
89229028
89233419
89242580
89243190
89243624
89244517
89244616
89244677
89246250
89246416
89246429
89247480
89248285
89255746
89260719
89260812
89260880
89262401
89262559
89262604
89263479
89263490
89264295
89269024
89273943
89274056
89274458
89274517
89275297
89276158
89277283
89277725
89278236
89278501
89279014
89280723
89280830
89281027
89282950
89283265
89283744
89284191
89284809
89285210
89288338
89288993
89290795
89292997
89293284
89294814
89295005
89295087
89300225
89300569
89300574
89300719
89301004
89301457
89301618
89301629
89302335
89302344
89302885
89303279
89303648
89303808
89307295
89314196
89314379
89314467
89314703
89315318
89315335
89315371
89316146
89316763
89316899
89317119
89317196
89317422
89317555
89318595
89319110
89319135
89320697
89321019
89321485
89321532
89321842
89321860
89322461
89322667
89322957
89322978
89323415
89323723
89323923
89324270
89324333
89325837
89326098
89326502
89326947
89328268
89328706
89328869
89330566
89331290
89331382
89331835
89332098
89332812
89333237
89333491
89334628
89335066
89335160
89335347
89335370
89335895
89337429
89338222
89339221
89339916
89340735
89340938
89341070
89341301
89341510
89343529
89343719
89346838
89346951
89346999
89347515
89347817
89349121
89349533
89350420
89350540
89351680
89351763

Supplementary Figure 3. - caption: LD matrix for selected SNPs on chromosome 15, displaying r^2 values from 0 (light gray) to 1 (dark red). SNP positions are plotted along the diagonal, and darker cells indicate stronger LD between variant pairs. Red arrow and diagonal red rectangles indicate the variant described by this study and the heatmap cells that show which variants are in LD with it. The other two variants in 100% LD with the described FANCI variant are also marked with red rectangles.
